# Biallelic *SYNE2* Missense Mutations Leading to Nesprin-2 Giant Hypo-Expression Are Associated with Intellectual Disability and Autism

**DOI:** 10.3390/genes12091294

**Published:** 2021-08-24

**Authors:** Natalie Young, Maria Asif, Matthew Jackson, Daniel Martín Fernández-Mayoralas, Mar Jimenez de la Peña, Beatriz Calleja-Pérez, Sara Álvarez, Eve Hunter-Featherstone, Angelika A. Noegel, Wolfgang Höhne, Peter Nürnberg, Boguslaw Obara, Muhammad Sajid Hussain, Iakowos Karakesisoglou, Alberto Fernández-Jaén

**Affiliations:** 1 Department of Biosciences, University of Durham, South Road, Durham DH1 3LE, UK; natalie.young@durham.ac.uk (N.Y.); mtjackson101@gmail.com (M.J.); eve.f.hunter-featherstone@durham.ac.uk (E.H.-F.); 2 Cologne Center for Genomics (CCG), University Hospital Cologne, University of Cologne, 50931 Cologne, Germany; maria-bukhari@hotmail.com (M.A.); wolfgang@fhoe.de (W.H.); nuernberg@uni-koeln.de (P.N.); 3 Center for Biochemistry, Medical Faculty, University of Cologne, 50931 Cologne, Germany; noegel@uni-koeln.de; 4 Department of Pediatric Neurology, Hospital Universitario Quirónsalud, 28223 Madrid, Spain; dmfmayor@yahoo.es (D.M.F.-M.); cataldo@telefonica.net (M.J.d.l.P.); 5 Pediatric Primary Care, C. S. Doctor Cirajas, 28017 Madrid, Spain; beacalleja@telefonica.net; 6 Department of Genomics and Medicine, Genomics and Medicine, NIMGenetics, 28108 Madrid, Spain; salvarez@nimgenetics.com; 7 Center for Molecular Medicine Cologne (CMMC), University Hospital Cologne, University of Cologne, 50931 Cologne, Germany; 8 School of Computing, Newcastle University, Newcastle upon Tyne NE4 5TG, UK; Boguslaw.Obara@newcastle.ac.uk; 9 Biosciences Institute, Newcastle University, Newcastle upon Tyne NE2 4HH, UK; 10 School of Medicine, Universidad Europea de Madrid, 28670 Madrid, Spain

**Keywords:** *SYNE2*, nesprin-2 giant, LINC complex, spectrin repeat 5, autism, intellectual disability, neurodevelopment

## Abstract

Autism spectrum disorder (ASD) is a group of neurological and developmental disabilities characterised by clinical and genetic heterogeneity. The current study aimed to expand ASD genotyping by investigating potential associations with *SYNE2* mutations. Specifically, the disease-causing variants of *SYNE2* in 410 trios manifesting neurodevelopmental disorders using whole-exome sequencing were explored. The consequences of the identified variants were studied at the transcript level using quantitative polymerase chain reaction (qPCR). For validation, immunofluorescence and immunoblotting were performed to analyse mutational effects at the protein level. The compound heterozygous variants of *SYNE2* (NM_182914.3:c.2483T>G; p.(Val828Gly) and NM_182914.3:c.2362G>A; p.(Glu788Lys)) were identified in a 4.5-year-old male, clinically diagnosed with autism spectrum disorder, developmental delay and intellectual disability. Both variants reside within the nesprin-2 giant spectrin repeat (SR5) domain and are predicted to be highly damaging using in silico tools. Specifically, a significant reduction of nesprin-2 giant protein levels is revealed in patient cells. *SYNE2* transcription and the nuclear envelope localisation of the mutant proteins was however unaffected as compared to parental control cells. Collectively, these data provide novel insights into the cardinal role of the nesprin-2 giant in neurodevelopment and suggest that the biallelic hypomorphic *SYNE2* mutations may be a new cause of intellectual disability and ASD.

## 1. Introduction

The LINC complex (linker of the nucleoskeleton and cytoskeleton) is a conserved macromolecular bridge, which spans the nuclear envelope in eukaryotes [1,2,3]. Members of the nesprin and SUN-domain family proteins in vertebrates interact in the nuclear envelope lumen, in a one-to-one stoichiometry forming the LINC complex core [4]. These direct associations enable the mechanical linkage of cytoskeletal filaments and associated structures, including the plasma membrane with the nuclear interior. Nesprins are members of the spectrin family of proteins, which exhibit a variable number of spectrin repeats, conserved cytoskeleton-binding domains and a C-terminal KASH-domain that integrates the proteins to the nuclear membrane [1,2,3,4,5,6,7,8]. The human genome contains four genes, termed *SYNE* (spectrin repeat-containing nuclear envelope protein) 1, 2, 3, and 4 which encode for nesprins (nuclear envelope spectrin repeat proteins) [5,9,10,11]. *SYNE1* and *SYNE2* in particular are highly complex and encode for a multitude of isoforms that differ in their length, domain composition, subcellular distribution and expression patterns [12]. The largest isoforms expressed by *SYNE1* and *SYNE2* are termed as giant, due to their massive molecular weight ranging from 0.8 (nesprin-2 giant) to 1 MDa (nesprin-1 giant). Besides their size, the main hallmark of the giant isoforms is the presence of an N-terminal actin-binding domain (ABD) which is composed of two calponin homology (CH) domains and the presence of a bulky spectrin repeat (SR)-containing segment which spatially separates the ABD from the C-terminal KASH-domain [6,7,13].

Although originally viewed solely as cytoskeletal adaptor proteins, increasing experimental evidence has transformed the initial perception. Regarding the functional aspects, nesprins are known to play fundamental roles in nuclear architecture and positioning [14,15,16,17,18,19], directed cell migration [20,21,22,23,24], cellular signalling [17,25,26], ciliogenesis [27,28,29], and mechanobiology [8,30,31,32,33,34,35,36]. These pivotal roles in cell biology and physiology are also evidenced in the significant phenotypes that are exhibited by nesprin transgenic and knockout murine models. The engineered genetic models exhibit phenotypic variance depending on the specific *SYNE* genomic segments that have been targeted. These, nevertheless, highlight important roles for both nesprin-1 and nesprin-2 in embryonic development, brain morphogenesis and development, retina formation, skeletal and cardiac muscle pathology, and skin tissue organisation [37]. Importantly, the biological and clinical significance of nesprins is further substantiated by their roles in health and disease in humans [13]. Proteins encoded by *SYNE1* are ubiquitously expressed, however, the strongest expression is detected within the cerebellum, skeletal and cardiac muscles [7], therefore, mutations that occur in this gene lead to neurological, neuromuscular, and musculoskeletal disorders. Mutations within *SYNE1* are currently linked to more diseases than *SYNE2*, these include; autosomal recessive cerebellar ataxia type 1 (ARCA1; OMIM #610743) [38,39], ataxia with amyotrophic lateral sclerosis (ALS)-like presentations [40,41], arthrogryposis multiplex congenita-3 (OMIM #618484) [42,43], Emery Dreifuss muscular dystrophy type 4 (OMIM #612998) [18,44] and dilated cardiomyopathy [45,46]. Moreover, *SYNE1* mutations may also contribute to intellectual disability [47], ALS [48,49], ASD [50,51] and bipolar disease [52,53,54,55,56].

Regarding disease pathogenicity of *SYNE2*, studies showed that heterozygous *SYNE2* mutations caused nuclear defects and associations with Emery-Dreifuss muscular dystrophy type 5 (OMIM #612999) [18,57]. Genetic studies in mice yielded different phenotypes depending on the implemented gene targeting strategy [37]. Nesprin-2 depletion in mice resulted in embryonic lethality [58]. However, the deletion of ABD-encoding exons affected nesprin-2 giant expression and increased epidermal thickness due to nuclear expansion without affecting the life span and the retina of the animals [15,59]. In contrast, the mouse pathogenic variant (c.13978C>T; p.Q4660*) also resulted in the absence of nesprin-2 giant, revealed (similar to *SYNE2* KASH-domain mouse knockouts [60]) significant roles of *SYNE2* in normal retinal development [61]. p.Q4660* caused migration defects of secondary neurons, failure of cone photoreceptors development, mislocalisation of rod photoreceptors and their premature apoptosis [61]. A more recent study associated *SYNE2* variants ((c.1721T>C (p.I574T; SR3), c.12001T>C (p.W4001R; SR35), c.12002G>A (p.W4001*; SR35) with the progression of DYT1 early-onset isolated dystonia [62].

Here, we describe a patient manifesting autism spectrum disorder (ASD) and developmental delay; whole-exome sequencing revealed compound heterozygous mutations in *SYNE2*. These mutations reside within the nesprin-2 giant SR5 domain. In silico approaches, including SR4-6 modelling, highlight the damaging nature of the identified mutations. Western blotting demonstrated a significant reduction in nesprin-2 giant protein levels in the patient cells, compared to parental levels. These findings support the notion that biallelic loss-of-function *SYNE2* mutations may cause a neurodevelopmental disorder characterised by intellectual disability and ASD.

## 2. Materials and Methods

### 2.1. Editorial Policies and Ethical Considerations

The study was carried out in accordance with the Declaration of Helsinki of the World Medical Association and approved by the Local Ethics Committees (Madrid, Spain; Ref. 30062019). Informed consent and written authorisation to publish clinical photographs of the patient were obtained from the parents, after a full explanation of the procedures.

### 2.2. Variant Identification of SYNE2

The already existing exome data of 410 trio cases were analysed to explore *SYNE2* (spectrin repeat-containing nuclear envelope protein 2) variants. Notably, all the patients considered manifested neurodevelopmental disorders of probable genetic origin. Pathogenicity of *SYNE2* variants was considered based on previous studies showing haploinsufficiency of this gene.

### 2.3. Genetic Study

Exome sequencing was performed using genomic DNA isolated (MagnaPure, Roche Diagnostics, Barcelona, Spain) from the whole blood of proband and parents. Libraries were prepared using the Ion AmpliSeq™ Exome Kit (Life Technologies, Waltham, MA, USA) and quantified by qPCR. The enriched libraries were prepared using Ion Chef™ and sequenced on PI™ Chip in the Ion Proton™ System (Life Technologies) to provide >90% of amplicons covered with at least 20×. Signal processing, base calling, alignment, and variant calling were performed on a Proton™ Torrent Server using the Torrent Suite™ Software. Variants were annotated using Ion Reporter™ Software, and pedigree analysis was performed using the Genetic Disease Screen (GDS) trio workflow. Variant filtering and prioritisation were performed with an in-house software program and a local database. The list of candidate variants was evaluated using previously published criteria [63]. Information from specific databases, including variants already described in association with a known phenotype (ClinVar [www.ncbi.nlm.nih.gov/clinvar/] accessed on 20 August 2021) and population frequency databases (dbSNP [https://www.ncbi.nlm.nih.gov/snp/?term=syne2] (accessed on 20 August 2021), gnomAD [https://gnomad.broadinstitute.org/] (accessed on 20 August 2021), 1000 Genome Project [https://www.internationalgenome.org/data/] (accessed on 20 August 2021), or NHLBI-ESP 6500 exomes [https://esp.gs.washington.edu/drupal/], accessed on 20 August 2021) to annotate variants that usually exist in the general population was used. We also estimated the pathogenicity of variants using CADD and a summation of selected prediction systems included in the dbNSFP database (SIFT, PolyPhen2, MutationTaster, MutationAssessor, LRT, FATHMM, and MetaSVM) for missense mutations. For mutations identified in splicing regions (including synonymous mutations), the effect on mRNA processing has been evaluated using the SpliceSiteFinder and MaxEntScan prediction systems, included in the SPiCE algorithm. Nucleotide position conservation has been evaluated according to the UCSC score ranges for the PhyloP tool. Finally, variant prioritisation was based on stringent assessments at both the gene and variant levels and taking into consideration the patient’s phenotype and the associated inheritance pattern. Candidate variants were visualised using IGV (Integrative Genomics Viewer). Candidate variants were evaluated based on stringent assessments at both, gene and variant levels, taking into consideration both the patient’s phenotype and the inheritance pattern. Variants were classified following the guidelines of the American College of Medical Genetics and Genomics (ACMG) [64]. A board of molecular clinical geneticists evaluated each variant classified as pathogenic, likely pathogenic, or variant of uncertain significance (VUS), and decided which, if any, had to be reported. In every case, causal variants were discussed with the referring physician and/or clinical geneticist. Identified variants were confirmed by Sanger sequencing.

### 2.4. Structural Modelling and Visualisation

Primary sequences corresponding to SR4-SR5-SR6 [65] of the wild-type, p.(E788K) and p.(V828G) mutant human nesprin-2 giant proteins (amino acids 576-932) were submitted to the PHYRE2 Protein Fold Recognition Server (http://www.sbg.bio.ic.ac.uk/phyre2; (accessed on 20 August 2021) [66]). The intensive modelling mode was used for all queries. Tertiary structures from obtained PDB coordinates were visualised as ribbon models using UCSF Chimera 1.14 [67]. Specific domains or residues were selected using the ‘Sequence’ tool and coloured appropriately. Individual side chains were depicted for residues of interest. The ‘MatchMaker’ function was utilised to superimpose wild-type and p.(V828G) mutant structures.

### 2.5. Lymphoblastoid (LCL) Cell Generation

We generated lymphoblastoid cell lines (LCLs) from the affected member and both of his parents, by using the previously described method [68]. In short, blood samples collected in lithium heparin tubes (BD Vacutainer^®^ PST™) were lysed in buffer (155 mM ammonium chloride, 10 mM potassium hydrogen carbonate and 0.1 mM disodium-EDTA) for 10 min. Immortalisation of lymphocytes was performed by Epstein-Barr-Virus and to remove T-lymphocytes, cyclosporine was used. This resulted in the generation of only B-lymphocytes.

### 2.6. Cell Culture

Lymphoblastoid cells were cultured in T75cm^2^ flasks (cytoOne, Starlab, Milton Keynes, UK). Cells were maintained in RPMI 1640 medium, supplemented with 10% Fetal Bovine Serum, 1% Penicillin/Streptomycin, 2 mM L-Glutamine, and 5% Briclone. All cell culture reagents were obtained from Gibco™ (Fisher Scientific, Loughborough, UK). The cultures were kept at 37 °C in a humidified atmosphere of 5% CO_2_.

### 2.7. Immunofluorescence (IF) 

The wild-type and patient LCL cells were seeded onto glass-coverslips (Scientific Laboratory Supplies (SLS), Wilford, UK; number 1.5) precoated with sterile 0.05 mg/mL Poly-D-Lysine solution. Two days post-seeding, coverslips were briefly rinsed with PBS (Phosphate-buffered saline) to remove cell culture media, and cells were fixed with 4% paraformaldehyde/PBS solution for 10 min, at room temperature. Following fixation, coverslips were washed twice with PBS (5 min incubations each) and then permeabilised with 0.5% Triton X-100/PBS solution for 10 min. Coverslips were then blocked for 10 min in PBG (1% BSA, 0.1% Fish gelatin solution in PBS; Sigma-Aldrich, Gillingham, UK), before being processed for indirect immunostaining. IF was performed in a humidified chamber by incubating the specimens with primary antibody (diluted in PBG) for 1 h, which was followed by 5 min washing steps repeated three times, each using PBS, and a 1 h incubation with the secondary fluorescently coupled antibody (diluted in PBG and in the presence of 2 μg/mL 4,6-diamino-2-phenylindone [DAPI; Sigma-Aldrich, Gillingham, UK] to stain nuclei). Finally, the coverslips were washed with PBG (two 10 min washes), followed by PBS (five washes, 5 min each), before they were mounted onto glass slides, using Vectashield anti-fade mountant medium H-1000 (Vector Laboratories, Peterborough, UK). Stained specimens were analysed using either a Zeiss LSM 880 with airyscan using identical imaging settings or a Zeiss Axioscope 40 upright fluorescence microscope mounted with a Zeiss AxioCam MRm camera.

### 2.8. Western Blotting (WB) Analysis 

LCL cells were collected, manually counted, and pelleted via centrifugation (5 min, 180 × g). The supernatants were aspirated, and the resulting cell pellets were dissolved in RIPA lysis buffer (50 mM Tris, 150 mM NaCl, 0.1% SDS, 1% Nonidet P-40, 0.5% Sodium-deoxycholate, 1% Protease Inhibitors (Protease Inhibitor Cocktail P2714, Sigma-Aldrich, Gillingham, UK), at a ratio of 200,000 cells per 10 μL of lysis buffer, and incubated on ice for 15 min. To aid protein extraction and shear DNA, lysates were passed 10 × through a 23 G needle (BD Microlance™, ThermoFisher Scientific, Loughborough, UK), before being centrifuged (13,000× *g*) for 10 min at 4 °C. Supernatants were collected, again passed 10 times through a 23G needle, and then mixed with sample loading buffer at a ratio of 1:5 (5 × Laemmli buffer, containing 5% 2-mercaptoethanol), before being denatured at 99 °C for 4 min. Proteins were separated through a 10% SDS-PAGE gel, and for proteins larger than 250 kDa, samples were separated through a NovexTM 4–12%Tris-Glycine gradient gel (Invitrogen). Cell lysate quality and equal protein loading were verified by staining the gels after SDS-PAGE with Expedeon InstantBlue protein stain following the manufacturer’s instructions (ThermoFisher Scientific, Loughborough, UK). For western blot analysis, proteins were transferred from the polyacrylamide gel to a methanol-activated PVDF membrane (Immobilon^®^-P, Merck Millipore, Burlington, MA, USA), using the Invitrogen semi-dry system (Power Blotter Station, ThermoFisher Scientific, Loughborough, UK). The semi-dry system utilises a typical transfer stack that consists of filter paper (Whatman 3MM) forming a bottom and a top layer, which encases a PVDF membrane (anode) and pre-run SDS-PAGE gel (cathode). The transfer stack was pre-soaked in Power Blotter 1-Step Transfer Buffer (1×; ThermoFisher Scientific, Loughborough, UK), before proteins were transferred using the pre-programmed method for high molecular weight proteins; constant amperage (1.3 A) for 10 min. Membranes were subsequently blocked for 30 min in 5% *w/v* non-fat dry milk solution in PBS, to prevent unspecific binding. Primary antibodies were diluted in 5% milk (PBS) and incubated overnight at 4 °C with agitation. Subsequently, membranes were washed three times in PBS-Tween (pH7.4, 0.1% Tween 20), and incubated with the appropriate horseradish peroxidase-conjugated secondary antibody for 1 h at room temperature. PVDF membranes were washed three times in PBS-Tween (pH7.4, 0.3% Tween 20) and three times in PBS-Tween (pH7.4, 0.1% Tween 20) before they were developed using the Clarity Western ECL Substrate (BioRad, Watford, UK) and then detected using the iBright 1500 imaging system (ThermoFisher Scientific, Loughborough, UK). The iBright is a digital western blot documentation system that provides signal linearity and equal or greater sensitivity to X-ray films. Densitometric analysis to determine relative nesprin-2 giant protein expression was carried out using Fiji. Data were normalised to GAPDH and then expressed as fold change compared to control, which was set as a value of 1.

### 2.9. Antibodies

Primary antibodies used: affinity-purified nesprin-2 CT (pAbK1, [14]), dilution 1:500 (IF) and 1:1500 (WB); mouse anti-nesprin-2 giant (clone K20-478, [6]), undiluted (IF); and mouse anti-GAPDH (Proteintech, Manchester, UK), dilution 1:10,000 (WB). Indirect IF analysis involved secondary chicken anti-Rabbit IgG conjugated Alexa Fluor 488, goat anti-Rabbit Alexa Fluor 568, and donkey anti-mouse Alexa Fluor 488 antibodies (ThermoFisher Scientific, Loughborough, UK, diluted 1:1000). Secondary antibodies implemented for western blot analysis include: goat anti-mouse IgG (whole molecule) Peroxidase antibody, dilution 1:10,000 and goat anti-rabbit IgG (whole molecule) Peroxidase antibody, dilution 1:5000 (Sigma-Aldrich, Gillingham, UK).

### 2.10. Statistical Analysis

GraphPad prism v9 (GraphPad, San Diego, CA, USA) was used to perform the statistical analysis. One-way ANOVA (Analysis of variance) with a Tukey’s post hoc test was used to determine statistical significance (*p* ≤ 0.05).

### 2.11. mRNA Isolation and qPCR

RNA was extracted from LCL cells using the RNeasy Mini kit (Qiagen, Hilden, Germany, #74104) which was further used to synthesise complementary DNA (cDNA) with the help of SuperScript II reverse transcriptase (RT) enzyme (Invitrogen, Carlsbad, CA, USA, #18064014). To test the effects of missense variants at the transcript level, we performed quantitative real-time PCR with cDNAs from the affected member and both parents. For quantification, gene-specific primers, two pairs, of *SYNE2* (Pair 1, Nes2G-F1: GCTTGCCCAGACTCTTTCTTGC and Nes2G-R1: GCCATCCCATTTCTCCAACTTGAC; Pair2, Nes2G-F2: TGCCTTGACATTC CTAA-GAAACCG and Nes2G-R2: GAGTCAACCACACTCACATCATCC) and *GAPDH* (Forward: TGACAACAGCCTCAAGATCATCAGCAA and Reverse: GTTTTTCTAGACGG-CAGGTCAGGTCCA) were mixed with PowerUp™ SYBR™ Green Master Mix (ThermoFisher Scientific, Loughborough, UK, #A25780) and the respective cDNA following the procedure described elsewhere [69].

## 3. Results

### 3.1. Clinical Features of the Patient

**Figure 1:**
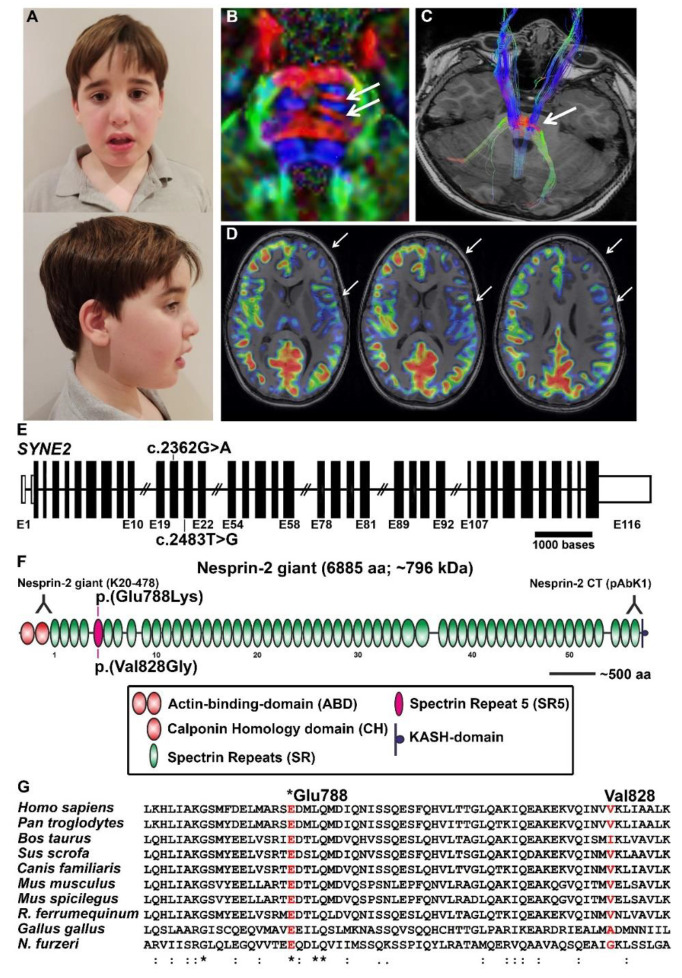
Clinical and genetic findings of a patient harbouring *SYNE2* genetic variations. (**A**) Frontal and side views of the patient at the age of 8 years, harbouring *SYNE2* mutations. (**B**) Diffusion tensor axial 2D view shows an intersection (arrows) of left transverse fibres (in red) into the corticospinal tract (blue fibres). (**C**) 3D Volumetric tractography. The mentioned projection fibres are superimposed on axial T1-weighted image. (**D**) Integrated registration of non-contrast perfusion-MRI (arterial spin labelling, ASL) and 3D-MPRAGE T1 weighted. Decreased cortical flow in the left hemisphere, mainly in the frontotemporal cortex, is noted. (**E**) Partial gene structure of human *SYNE2* (NM_182914.3) showing selected coding exons as vertical black bars and non-coding exons as white bars (both drawn according to the indicated scale), whereas horizontal line shows introns with arbitrary length. Identified variants lie in exons 20 and 21 as indicated. (**F**) Nesprin-2 giant protein structure consisting of 6885 amino acids with an estimated size of 796 kDa. Colour codes for each domain is indicated in the box located at the bottom of the image. Identified mutations are shown on the top and bottom of spectrin repeat 5 (SR5, shown in purple). The epitopes of the nesprin-2 antibodies are shown at the N-terminus and the C-terminus of the protein (inverted Y). (**G**) Multiple alignments of nesprin-2 sequences from species indicated at left, performed by Clustal Omega showing high (Glu788) and modest (Val828) conservation in vertebrates. Due to space constraints, short forms of greater horseshoe bat (*Rhinolophus ferrumequinum*) and turquoise killifish (*Nothobranchius furzeri*) are written. *: conserved amino acid residues.

A 4.5-year-old male, the first child from non-consanguineous healthy young parents of Spanish origin (Figure 1A), was referred to our clinic. The patient was born via an uncomplicated vaginal delivery at 39-weeks of gestational age. Birth weight was 3.00 kg (15th centile). Global developmental delay was noted in the first month of life. He walked unsupported at 15 months; at the age of 3 years, he started using some disyllabic words although showed no interest in interacting with other children. This is exemplified by the fact that he always played alone. His social eye contact was minimal.

The clinical examination conducted at the age of 4.5-years disclosed a weight of 22 kg (95th centile), a height of 115 cm (95th centile), and an OFC of 54 cm (95th centile), without dysmorphic features. Observation of the patient revealed verbal and nonverbal communication deficits, severe hyperkinetic behaviour, and stereotyped movements. Routine laboratory screening including thyroid function and neurometabolic tests were within normal range. Sleep video-EEG test and auditory evoked potentials displayed normal results. Brain 3T MRI (Magnetic Resonance Imaging) did not reveal any significant structural malformations; a slight and unspecified cortical hypoperfusion of the left hemisphere was observed compared with the contralateral hemisphere (Figure 1B–D). Conventional genetic studies (karyotype, array comparative genomic hybridisation analysis and Fragile X studies) also revealed no abnormalities. At the age of 5 years, this child still displayed severe verbal and nonverbal communication deficits. Neuropsychological assessments to measure intellectual functioning and development, as well as language abilities, were conducted. Specifically, the following tests and scales were used: the Leiter International Performance Scale (Leiter-3), the Peabody Picture Vocabulary Test, Third Edition-PPVT-III-, and the Battelle Developmental Inventory. It was not possible to perform the WPPSI test due to his severe language and communication problems. Notably, Leiter-3 revealed the presence of an IQ (Intelligence Quotient) of 70. PPVT-III revealed a vocabulary level below the 5th centile. Battelle Neurodevelopment Inventory showed scores below 5th centile in adaptive, personal-social, communication and cognitive domains. Further information was obtained using the Autism Diagnostic Interview-Revised (ADI-R), the Autism Diagnostic Observation Scale (ADOS) and the Vineland Adaptive Behaviour Scales-3. All his ADI-R and ADOS algorithm scores were above autism cut-off items. A total Vineland score of 55 revealed his severe adaptive problems, with particularly lower scores in the communication, daily living skills and socialisation domains. In summary, the patient met the criteria for intellectual disability and autism.

### 3.2. Genetic Results 

Whole-exome trio analyses revealed compound heterozygous variants in *SYNE2*. A missense variant NC_000014.8: g.64457670T>G, NM_182914.3; c.2483T>G; p.(Val828Gly) and a missense variant NC_000014.8: g.64457177G>A, NM_182914.3; c.2362G>A, p.(Glu788Lys), were identified (Figure 1E). Segregation analysis confirmed a compound heterozygous state, as both variants were identified in heterozygosity in the mother (p.(Glu788Lys)) and father (p.(Val828Gly)), consistent with an autosomal recessive inheritance. These mutations were further confirmed by Sanger sequencing. Furthermore, his healthy brother was heterozygous only for the p.(Val828Gly). Both mutations have been described in heterozygosity in international databases, as 1000 Genomes Project and gnomAD exomes/genomes (frequency < 0.001%). None of them has been described in homozygosity in these databases. Pathogenicity prediction of *SYNE2* variants is described in Table 1.

### 3.3. Position of SYNE2 Mutations within Nesprin-2 giant and Predicted Consequences

The two identified mutations (p.(Glu788Lys) and p.(Val828Gly)) are situated in the nesprin-2 giant spectrin repeat (SR) domain 5 (SR5, AS 735-838). Nesprin-2 giant is the largest isoform of *SYNE2* harbouring in total 56 SRs (Figure 1F; [65]). An SR domain is composed of three alpha-helices (A, B, and C) [70]. A multiple sequence alignment containing nesprin-2 giant SR5 sequences from different vertebrates shows strong conservation of the Glu788 position and modest conservation of Val828 (Figure 1G).

**Figure 2:**
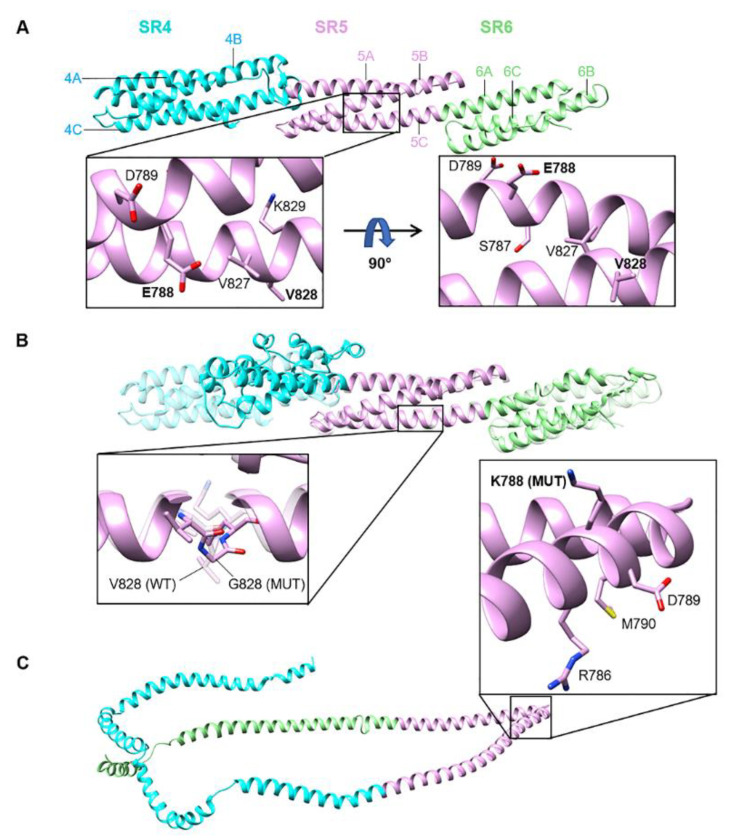
Structural models of spectrin repeats SR4-SR5-SR6 for wild-type and mutant nesprin-2 giant proteins. (**A**) Wild-type (WT) SR4-SR5-SR6 with major helices labelled. WT residues within the affected region of SR5 (helices 5B and 5C) are presented in two orientations (90°-rotated; inset). Residues E788 and V828 (altered in mutants) are shown in bold. (**B**) Superposition of V828G mutant (solid) and WT (transparent) SR4-SR5-SR6 structures. SR4 and helix 6B (SR6) conformations are distorted in the V828G mutant, whilst SR5 is structurally homologous to WT. The peptide backbone (as well as residue side-chains) is presented in ‘stick’ form for positions 827–829 to distinguish between mutant glycine and WT valine at position 828 (inset). G828 mutant (MUT) and V828 (WT) residues (labelled) are depicted as solid and transparent structures, respectively. (**C**) E788K mutant SR4-SR5-SR6, showing complete loss of the typical triple-helical SR structure. K788 (MUT) and neighbouring residues are shown (inset). A superposition with the WT structure was not possible, due to extreme conformational differences. All structural models were predicted using PHYRE2. Stick representations are coloured by element (bound hydrogens are not depicted): carbon = pink, nitrogen = blue, sulfur = yellow, oxygen = red. For all structures, SR4 = turquoise, SR5 = pink, SR6 = green.

An inspection of a spectrin structure (3fb2, human SPTAN1 alpha-chain, spectrin domains 12 and 13, with comparatively low sequence homology to the nesprin-2 giant spectrin domain 5) likely shows equivalent positions for Glu788 a salt bridge with a lysine and a hydrophobic contact with Val828, stabilising the interaction of adjacent helices in the structure; a p.Glu788Lys exchange with its electrostatic charge inversion may severely disturb the helix/helix interaction, whereas the p.(Val828Gly) exchange should not dramatically influence the structure and thus the function, but certainly causes higher flexibility around this position, and thus may slow down the folding process or enhances the proteolytic accessibility. In agreement, structural modelling using PHYRE2 of the nesprin-2 giant SRs 4,5,6 (Figure 2A) indicates that p.(Val828Gly) (SR5, helix C) leaves the SR5 structure largely unaffected, whereas structural effects were predicted for the flanking SR4 and SR6 domains (Figure 2B). In contrast, the p.(Glu788Lys) (SR5, helix B) mutation is predicted to be highly disruptive and to affect the folding of the entire SR4-6 segment (Figure 2C).

### 3.4. Expression and Functional Results 

**Figure 3:**
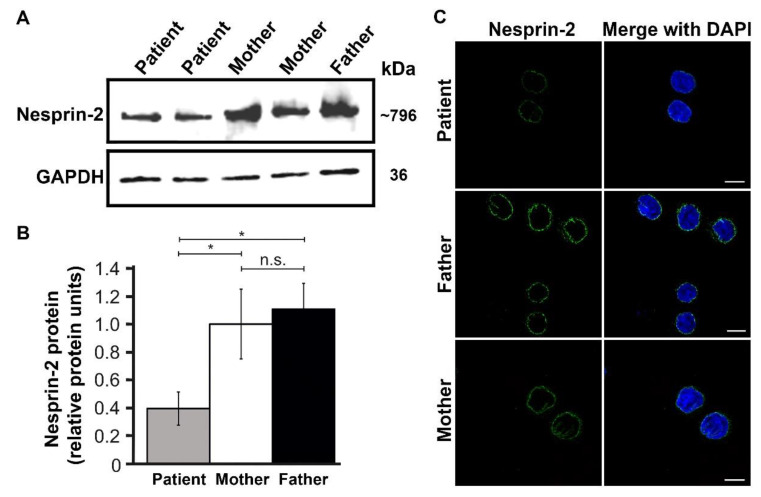
The compound heterozygous *SYNE2* mutations p.(Glu788Lys) and p.(Val828Gly), affect the expression of nesprin-2 giant but not the subcellular distribution of nesprin-2 to the nuclear envelope. (**A**) A representative western blot was performed on cell lysates of LCLs from the patient and both parents showing a reduced quantity of nesprin-2 giant protein in the patient (upper panel), GAPDH was used as an internal control (lower panel). (**B**) Comparative quantification of nesprin-2 giant showing a 0.6-fold decrease in the patient (grey bar) as compared to the mother (white bar). Signal intensity was quantified by densitometry. Values are the mean of six independent experiments, error bar represents S.E.M. (standard error mean). Statistical significance was assessed using a one-way ANOVA with Tukey’s post hoc test, * *p* < 0.05; n.s. = non-significant. (**C**) Confocal microscopy images of LCLs immunolabelled for nesprin-2 (green) of parents (middle and lower panel) and patient (upper panel) showing a marked reduction in the patient. Scale bar 10 µm.

To gain functional insights into the pathogenicity of the *SYNE2* mutations, LCLs derived from the patient and the parents were subjected to immunoblotting and immunocytochemistry. In comparison with paternal and maternal LCLs, a strong reduction of nesprin-2 giant levels in the patient was indicated by immunoblotting (Figure 3A). Quantitative analysis of the western blot data demonstrated a significant reduction by ~60% in nesprin-2 giant expression in the patient when compared to maternal control (Figure 3B). Confocal microscopy of nesprin-2 stained LCLs under identical staining and imaging conditions confirmed this finding, as the staining was less intense in the patient LCLs. Nevertheless, the nuclear envelope localisation of nesprin-2 remained unaffected in the patient LCLs (Figure 3C; Supplemental Figure S1). In addition, abnormal aggregates of the mutant nesprin-2 proteins or nuclear shape deficits were not evident in the patient LCLs (Figure 3C; Supplemental Figure S1). Moreover, real-time PCR analysis of the nesprin-2 giant mRNA levels did not reveal any significant changes in *SYNE2* transcription (Supplemental Figure S2). In summary, the data indicates that the *SYNE2* biallelic mutations affect the stability of nesprin-2 giant and thus reduces the protein levels, but *SYNE2* transcription and nesprin-2 giant localisation in the patient remains largely unaffected.

## 4. Discussion

*SYNE2* missense mutations in the heterozygous state are frequent; 95% of these variants are benign. The pLI [71] and the haploinsufficiency scores [72] of *SYNE2* are 0.0 and 52.87 respectively, and the Z score for missense variations is −1.45 [73]. According to these data, homozygosity or compound heterozygosity states is probably necessary to alter the expression of the protein and therefore its functions. In agreement, the current study reports two novel compound heterozygous *SYNE2* missense mutations (p.(E788K) and p.(V828G)), which show an association with autism spectrum disorder (ASD). In comparison to parental control LCLs, the analyses of the patient indicated that the p.(E788K) and p.(V828G) mutations do not affect the nuclear targeting of mutant proteins. This is not surprising based on the consideration that the mutations reside within the nesprin-2 giant N-terminal half (SR5) and not in the C-terminal KASH domain, which mediates nuclear envelope targeting [1,2,6]. Although *SYNE2* transcript expression in the patient LCLs is unaffected, the amount of nesprin-2 giant protein is significantly reduced. This finding highlights that the mutations, and in particular SR5 biology, play a key role in the stability of the entire nesprin-2 giant molecule. It is noteworthy that LCL nuclei are mostly spherical, occupying the majority of the cytoplasm and are presumably under less mechanical strain as the cells are weakly adherent to the substratum [68]. Therefore, the effects on nesprin-2 levels in adhesive patient cells, such as in the brain, may be more pronounced due to increased pulling forces on the nesprin-2 molecules. In keratinocytes, the stiffness of the extracellular environment affects cell spreading (colony density) and has profound effects on LINC complex protein expression [36]. To gain molecular insights into the underlying regulatory mechanisms, the identification of SR5 interacting molecules, and the study of SR5 mechanobiology is essential. Furthermore, the establishment of induced pluripotent stem cells from the patient and the consecutive generation of relevant neuronal cells may be more suitable to assess holistically the functional consequences of nesprin-2 giant mutations (p.(E788K) and p.(V828G)) on cellular architecture and physiology. Considering the oligomerisation properties of other members of the spectrin family [74] it is plausible that the SR5-containing region may function as a structural node or an oligomerisation domain, which stabilises nesprin-2 giant structure and thus may affect protein turnover. It is also worth mentioning that impaired spectrin family member functions are associated with several genetic disorders where respective mutations, located within spectrin repeats, result in the instability of the protein, thus responsible for the disease phenotype [75,76]. On these grounds, we propose the hypomorphic effect of our *SYNE2* mutations features phenotypic variability.

Neurodevelopmental disorders are etiologically diverse. Sequencing studies have demonstrated numerous mutations that cause brain dysfunction, implied in these neurodevelopmental disorders. Although 60% of these mutations follow autosomal-recessive inheritance [77], de novo heterozygous mutations and copy number changes explain the majority of cases with intellectual disability or autism in non-consanguineous populations [78,79]. Rare disorders caused by non-recurrent mutations or sporadic biallelic mutations are persistently challenging to investigate, just as our case has demonstrated. 

ASD is characterised by impaired social interactions, restrictive interests, and repetitive behaviours. A candidate gene can be identified in 40% of individuals diagnosed with ASD, increasing the knowledge of the mechanisms involved in normal neurodevelopment [80,81]. However, for a minimum of 50% of genes, the impact in humans has not yet been described [82]. Despite the entries of more than 200 OMIM phenotypes per year [83], more than 1000 genes associated with developmental disorders are still not validated [84]. According to haploinsufficiency scores, 17% of the human genes are likely to be haploinsufficient; of these genes, 70% have no known phenotype [71]. This unawareness might be extrapolated to the haplosufficient genes; the presence of unknown autosomal recessive disorders could be underlying ASD, as supported by studies in populations with a high consanguinity [85].

Previously described ASD-linked genes are related to complex processes or pathways associated with neuronal growth and migration, axon guidance, synaptic organisation, chromatin regulation, channel activity, or developmental signalling pathways such as Wnt [86,87,88]. Different neuroanatomical alterations have been reported in patients with ASD, including disorganisation in the neocortex, abnormal neuronal subtype distribution or connectivity anomalies [86]. Autism is a complex disease (i.e., it is not a monogenic disorder) and combined with the multi-functionality of the nesprin-2 giant protein, we anticipate a complex underlying molecular and cellular pathology.

*SYNE2* variations may affect *SYNE1* functions, which has already been associated with intellectual disability [47] and ASD [50,51]. Previous studies have shown that both nesprin-1 and nesprin-2 giant molecules compete for nesprin-3 binding [89]. Therefore, the reduction of nesprin-2 giant protein levels may have collateral effects on LINC complex composition, and structure, including the cytoskeletal landscapes of the outer nuclear membrane in patient cells. Nesprins mediate neurogenesis, neuronal migration, axon termination, and synapse formation, dynamic processes that rely on properly functioning cytoskeletons [20,89,90,91]. This cardinal role in neurogenesis and/or regulation of Wnt-signalling [26,91] may be underlying the previously suggested association of *SYNE2* with ASD or intellectual disability [92,93]. Identifying additional *SYNE1* and/or *SYNE2* mutations that show associations or direct involvement in autism will aid to elucidate the underpinned molecular mechanisms. Specifically, it will be instrumental to evaluate the consequences of nesprin-2 giant downregulation on neurite cytoskeleton organisation, and motor protein distribution, in order to evaluate their effects on neuronal cell structure, nuclear movement and dendrite formation/dynamics. Although a possible relation between *SYNE2* and ASD had been previously suggested [92,93], our results clearly support this relationship, particularly after the functional analyses of described mutations in a patient with ASD.

At the methodology level, the current study also demonstrates the successful identification of the nesprin-2 giant using a power blotter, which significantly reduces the detection of this massive ~796 kDa protein to a few hours, compared to existing labour-intensive and time-consuming western blotting techniques [94]. In addition, the data generated indicates that nesprin-2 is strongly expressed in lymphoblastoid cells and importantly exclusively confined to the nuclear envelope. This finding is pivotal because it simplifies and restricts the analysis of nesprin-2 mutations to the nuclear envelope in LCL cells. In other cellular models, the study of nesprin-2 biology is hampered by the presence of a multitude of isoforms that occupy additional subcellular locations besides the nucleus [13,37]. As LCL cells generation is cost-effective and less invasive, we posit that the presence of nesprin-2 in LCL cells will facilitate the study of *SYNE2*-associated pathomechanisms and elucidate in general, the role of LINC complex proteins in human diseases.

## 5. Conclusions

We have identified novel compound heterozygous missense variations in *SYNE2*, which consequently reduce the amount of nesprin-2 giant and thus, hypomorphic variations as the cause of ASD and intellectual disability.

## Figures and Tables

**Table 1:** Pathogenicity prediction of *SYNE2* (NM_182914.3) variants by various in silico tools.

Tool	c.2362Gv>vA; p.v(Glu788Lys)	c.2483Tv>vG; p.v(Val828Gly)
PANTHER	Neutral (0.128)	Disease causing (0.723)
PhD-SNP	Disease causing (0.886)	Disease causing (0.862)
SIFT	Disease causing (0.000)	Disease causing (0.000)
SNAP	Disease causing (0.595)	Disease causing (0.555)
Meta SNP	Disease causing (0.681)	Disease causing (0.681)
Provean	Neutral (−2.272)	Disease causing (−3.649)
MuPro	Decrease protein stability (delta delta G: −1.14)	Decrease protein stability (delta delta G: −2.32)
SNPs and GO	Neutral	Neutral
Polyphen-2	Probably damaging (0.999)	Possibly damaging (0.465)
Mutation Taster	Polymorphism (56)	Polymorphism (109)
CADD score	27.2	13.2
FATHMM	Tolerated (0.58)	Tolerated (0.72)
ACMG Interpretation	Uncertain significance (PM2)	Uncertain significance (PM2)

Note: Pathogenicity prediction scores are given by PANTHER, PhD-SNP, SNAP, and Meta-SNAP is shown in the table; here, the scale is between 0 and 1 where more than 0.5 score signifies disease-causing variant. In the case of SIFT, positive values: more than 0.5 shows neutral effects of mutation. MuPro predicts the structural stability of protein where a score less than 0, signifies decreased stability of the mutant protein. SNPs and GO determine the pathogenic effects of variants based on vector machines, scoring with accuracy = 82% and Matthews correlation coefficient = 0.63. Mutation Taster scores may range from 0.0 to 215, the greater the score the more pathogenic is the variant. CADD score of more than 20 shows a pathogenic variant, whereas beyond 30 it indicates high pathogenicity. FATHMM suggests positive values for the tolerated mutations in a specific region, however, the negative values mean intolerance or damaging mutations. ACMG Interpretation describes pathogenic”, “likely pathogenic”, “uncertain significance”, “likely benign”, and “benign” variants based on evidence from population, computational, functional, and segregation data.

## Data Availability

Associated data of this study could be requested from corresponding authors.

## Supplementary Materials

The following are available online at https://www.mdpi.com/article/10.3390/genes12091294/s1, Figure S1: The nesprin-2 subcellular localisation is unaffected in parental and patient LCL cells. Figure S2: Nesprin-2 giant transcript levels are not perturbed in the patient cells.
