# Supplementary material for: Biallelic SYNE2 Missense Mutations Leading to Nesprin-2 Giant Hypo-Expression Are Associated with Intellectual Disability and Autism
Source: Genes (Basel). 2021 Aug 24;12(9):1294. doi: 10.3390/genes12091294 (PMC8470961; doi:10.3390/genes12091294)
Supplement: Supplementary file 1 [file genes-12-01294-s001.zip › genes-1300971-supplementary.pdf]

Supplemental Figures

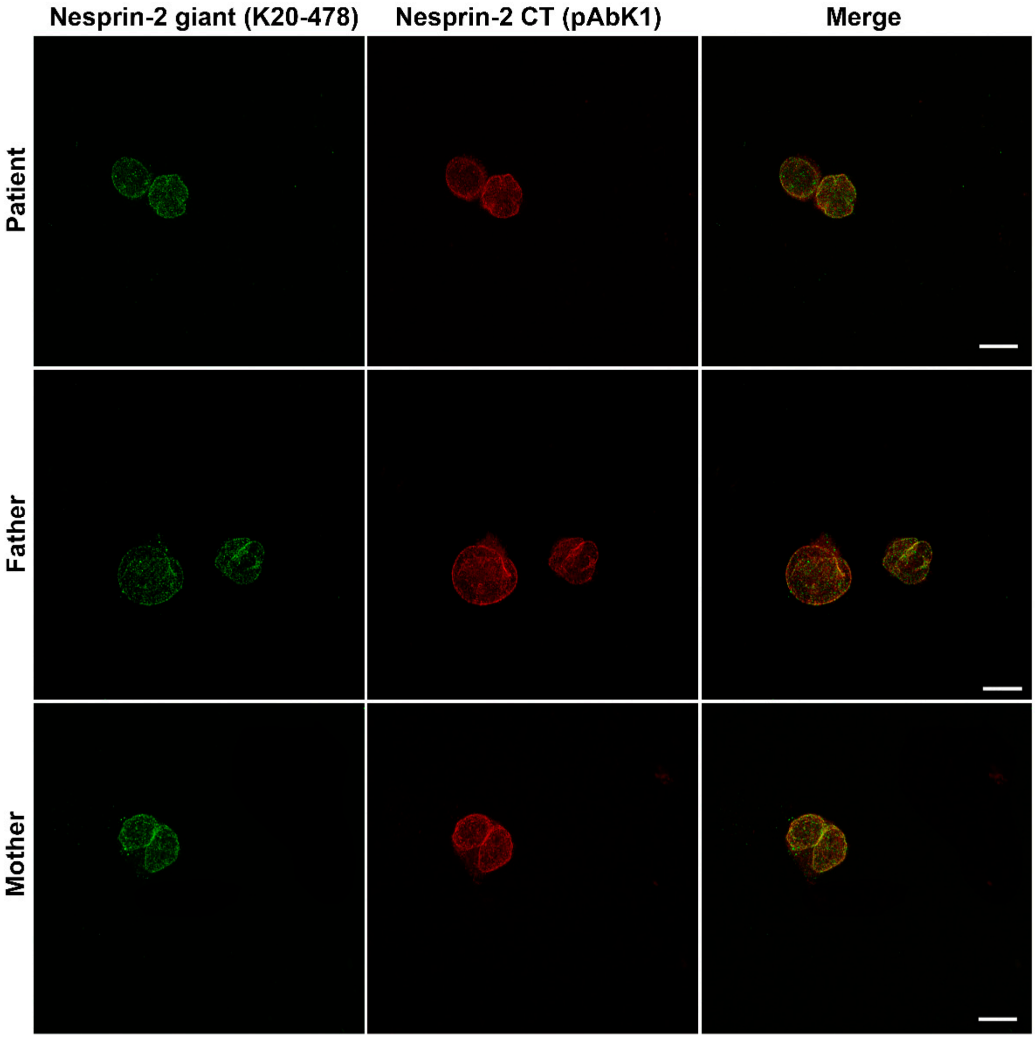

Figure S1. Fluorescent microscopy images of LCL cells derived from the affected family member and his parents stained with mouse monoclonal anti-nesprin-2 giant (K20-478; ABD-epitope; green) and rabbit polyclonal nesprin-2 CT (pAbK1; red), which has the epitope at the C-terminus of nesprin-2 giant. Scale bar, 10 μm.

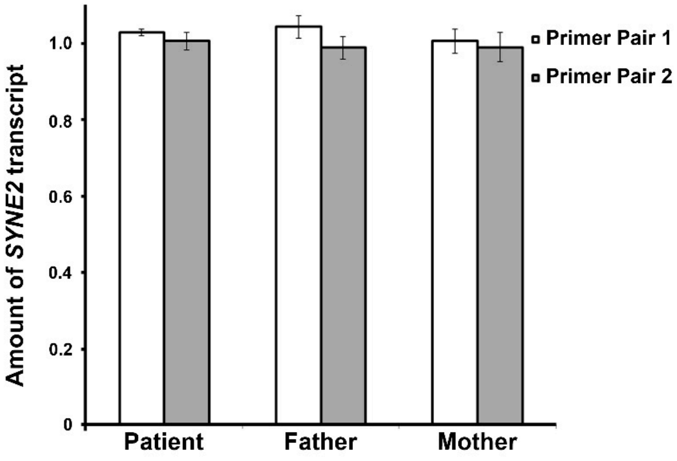

Figure S2. Relative mRNA amount of *SYNE2* measured in the patient and both his parents by using two different sets of nesprin-2 giant specific primers. Error bars represent SD (standard deviation). Experiment was performed three times. No significant difference in the amount of mutant mRNA compared to both parents is noted.
